# Ascensão Matinal da Pressão Arterial e Obesidade em Pacientes Hipertensos

**DOI:** 10.36660/abc.20230608

**Published:** 2023-10-26

**Authors:** Sayuri Inuzuka, Weimar Kunz Sebba Barroso

**Affiliations:** 1 Programa de Pós-Graduação em Ciências da Saúde Faculdade de Medicina Universidade Federal de Goiás Goiânia GO Brasil Liga de Hipertensão – Seção Cardiovascular e Programa de Pós-Graduação em Ciências da Saúde - Faculdade de Medicina da Universidade Federal de Goiás, Goiânia, GO – Brasil; 2 Hospital Israelita Albert Einstein Goiânia GO Brasil Hospital Israelita Albert Einstein, Goiânia, GO – Brasil

**Keywords:** Hipertensão, Obesidade, Monitorização Ambulatorial da Pressão Arterial

A obesidade e a hipertensão são duas das doenças mais comuns que estão geralmente interrelacionadas. Estima-se que os cinco principais fatores de risco modificáveis – dislipidemia, diabetes, hipertensão, obesidade e tabagismo sejam responsáveis por mais de 50% da mortalidade cardiovascular.^1^ Há muitos pacientes hipertensos com lesões subclínicas nos estágios iniciais da doença que em geral não são identificadas pelo modelo de avaliação tradicional.^2 , 3^ A presença de hipertrofia ventricular esquerda (HVE) determina a estratificação de risco geral e é um importante alvo terapêutico na hipertensão. Tanto a obesidade como a hipertensão podem ter efeitos adicionais e interativos sobre a HVE.^4^ Para identificar lesão precoce no sistema cardiovascular, exames complementares mais específicos têm sido recomendados por diretrizes de hipertensão.^5 - 7^

Diferentes métodos têm sido propostos para determinar a ascensão matinal da pressão arterial (AMPA),^8 - 11^ mas ainda existem várias lacunas quanto ao estabelecimento de pontos de corte e recomendações de significância clínica.^12^ Alguns estudos mostraram que a AMPA tem um valor prognóstico adverso, independentemente da média da pressão arterial de 24 horas.^7^ Contudo, dada a dificuldade de se padronizar o cálculo desse parâmetro, o valor preditivo incremental ainda não está claro, e sua baixa reprodutibilidade indica que, de acordo com as diretrizes, a AMPA não deve ser usada na prática clínica.^5 , 7^

Tanto a AMPA como a obesidade mostraram-se estar associadas com HVE em um estudo transversal com 203 pacientes hipertensos nesta edição dos Arquivos Brasileiros de Cardiologia, Palmeira et al.^13^ Os pesquisadores da EPM/UNIFESP - Escola Paulista de Medicina da Universidade Federal de São Paulo encontraram uma AMPA de 16mmHg como limiar associado com HVE no grupo obeso, e o limiar de 22mmHg no grupo não obeso.

Um grande estudo longitudinal com 2020 pacientes e 19,7 anos de acompanhamento sugere que a inclinação da regressão linear de medidas da Pressão Arterial Sistólica (PAS) em intervalos de tempo no descenso do sono (calculado como a diferença entre a PAS matinal e a PAS mais baixa à noite) poderia identificar independentemente indivíduos com risco cardiovascular aumentado.^8^ O estudo J-HOP calculou a AMPA como uma variável contínua e mostrou sua associação independente com ocorrência de acidente vascular cerebral.^9^ Outro estudo de Kario et al.^10^ sugere que a inclinação da ascensão matinal reflete a taxa de mudança da pressão arterial do período noturno para a manhã, e mostrou que a atenuação dessa inclinação pode indicar possíveis benefícios.^10^ No estudo discutido neste editorial, a ascensão matinal foi calculada como a diferença entre a PAS matinal e a PAS mais baixa durante o sono. Os autores também estabeleceram pontos de corte para a AMPA que se correlacionaram com HVE em pacientes hipertensos obesos e não obesos.

De acordo com as principais diretrizes de hipertensão, testes complementares mais específicos para análise de biomarcadores são usados para a identificação precoce de dano cardiovascular.^5 , 7^ A identificação de pacientes em maior risco permite melhor tratamento, menos eventos cardiovasculares, e melhor qualidade de vida. Biomarcadores cardiovasculares são importantes na medicina de precisão. A definição de pontos de corte é importante para a aplicação desses valores na nossa rotina.
