# Supplementary material for: Ascensão Matinal da Pressão Arterial e Obesidade em Pacientes Hipertensos
Source: Arq Bras Cardiol. 2023 Oct 26;120(9):e20230608. [Article in Portuguese] doi: 10.36660/abc.20230608 (PMC12080713; doi:10.36660/abc.20230608)
Supplement: Translation

# Translation: Morning Surge Blood Pressure and Obesity in Hypertensive Patients

**Authors:** Sayuri Inuzuka, Weimar Kunz Sebba Barroso

**Affiliations:** 1 Brasil Liga de Hipertensão – Seção Cardiovascular e Programa de Pós-Graduação em Ciências da Saúde - Faculdade de Medicina da Universidade Federal de Goiás, Goiânia, GO – Brasil; 2 Brasil Hospital Israelita Albert Einstein, Goiânia, GO – Brasil

**Keywords:** Hypertension, obesity, ambulatory blood pressure monitoring

Obesity and hypertension are two of the most common diseases and they are usually interrelated. The five main modifiable risk factors – dyslipidemia, diabetes, hypertension, obesity and smoking are estimated to account for more than 50% of cardiovascular mortality.^1^There are many hypertensive patients with subclinical lesions in the early stages of the disease that are not generally identified by the traditional assessment model.^2 , 3^The presence of left ventricular hypertrophy (LVH) determines general risk stratification and is a major therapeutic target in hypertension. Both obesity and hypertension may have additive and interactive effects on LVH.^4^In order to identify early cardiovascular system damage, more specific complementary exams for biomarker analysis have been recommended by hypertension guidelines.^5 - 7^

Different methods to determine morning surge blood pressure (MSBP) elevation have been proposed,^8 - 11^but there are still several gaps regarding the establishment of cutoffs and clinical meaningful recommendations.^12^Some studies showed that MSBP have an adverse prognostic value, independently of 24h mean blood pressure.^7^However, due to the difficulty in standardizing the calculation of this parameter, the incremental predictive value is still unclear and its low reproducibility indicate that, according to guidelines, it should not be used in clinical practice.^5 , 7^

Both MSBP and obesity have been shown to be associated with LVH in a cross-sectional study of 203 hypertensive patients in this issue of *Arquivos Brasileiros de Cardiologia* , Palmeira et al.^13^The researchers of the EPM/UNIFESP - Escola Paulista de Medicina da Universidade Federal de São Paulo have found elevated morning surge of 16mmHg as a threshold associated with LVH in the obese group, and the threshold of 22mmHg in the non-obese group.

A large longitudinal study with 2020 patients, 19.7-year follow-up, suggests that the rate of sleep-trough MS. The rate of STMS was derived as the slope of linear regression of sequential systolic blood pressure (SBP) measures on time intervals within the sleep-trough (calculated as the difference between morning SBP and lowest nighttime SBP) could identify independently subjects with an increased cardiovascular risk.^8^The *J-HOP study* calculated MSBP as a continuous variable and showed that it was independently associated with stroke events.^9^Another study by Kario et al.,^10^suggests that the morning surge slope reflects the rate of change of blood pressure from night time to morning, and that the attenuation of this slope may indicate possible benefits.^10^In the study discussed in this editorial, morning surge was calculated as the difference between morning SBP and the lowest SBP during sleep. The authors also established MSBP cutoff points that were correlated with LVH in obese and non-obese hypertensive patients.

According to the main hypertension guidelines, more specific complementary tests for biomarker analysis are used for the early identification of cardiovascular damage.^5 , 7^The identification of patients at higher risk allows better treatment, fewer cardiovascular events and better quality of life. Cardiovascular biomarkers are important in precision medicine. The definition of cutoff points is important for the usability of these values in our routine.
